# Respiratory health and eruptions of the Nyiragongo and Nyamulagira volcanoes in the Democratic Republic of Congo: a time-series analysis

**DOI:** 10.1186/s12940-020-00615-9

**Published:** 2020-06-05

**Authors:** Caroline Michellier, Patrick de Marie Chimusa Katoto, Michèle Dramaix, Benoit Nemery, François Kervyn

**Affiliations:** 1 Natural hazards service, Department of Earth Sciences, Royal Museum for Central Africa, Leuvensesteenweg 13, 3080 Tervuren, Belgium; 2 Centre for Environment and Health, Department of Public Health and Primary Care, KU Leuven, Herestraat 49, 3000 Leuven, Belgium; 3 Department of Internal Medicine, Faculty of Medicine, Catholic University of Bukavu, Bugabo 2, Av. de la Mission, Commune de Kadutu, Bukavu, Democratic Republic of Congo; 4 Research Centre of Epidemiology, Biostatistics and Clinical Research, School of Public Health, Université Libre de Bruxelles, Campus Erasme, Route de Lennik 808, 1070 Brussels, Belgium

**Keywords:** Ambient air pollution, Acute respiratory symptoms, Spatial analysis, Active volcanoes, Nyamulagira-Nyiragongo, Goma, Democratic Republic of Congo

## Abstract

**Background:**

Nyamulagira and Nyiragongo are active volcanoes situated close to Goma (North Kivu, Democratic Republic of Congo). These volcanoes are among the most prolific sources of volcanic SO_2_ pollution on earth.

**Objective:**

We investigated the possible spatiotemporal relationships between volcanic degassing represented by eruptive emissions of SO_2_ that occurred between 2000 and 2010, and the incidence of acute respiratory symptoms (ARS) in populations living in areas up to more than 100 km from the volcanoes.

**Methodology:**

The total flux of SO_2_ emitted during eruptions since 2000 and the average spatial distribution of the volcanic plume (2004–2008) were based on publicly available remote sensing data. The monthly numbers of adults and children reporting acute respiratory symptoms were extracted from health data collected routinely by selected local health centres and hospitals between 2000 and 2010. The monthly numbers of persons with ARS recorded during or after eruptions were compared with those recorded before eruptions, using negative binomial regression models allowing the calculation of incidence rate ratios (IRR) and their 95% confidence intervals. We first compared years with and without eruptions and then considered shorter time-windows (months).

**Results:**

In the investigated area, ARS were the second most frequent cause of medical visits (12.2%, *n* = 3.2 million cases), after malaria (32.3%, *n* = 8.4 million cases). SO_2_ emissions gradually increased 30 to 50 times in 2010 compared to 2002. Taking 1999 as a reference, the IRR for ARS increased three-fold between 2000 [0.9 (0.8, 1.1)] and 2009 [2.8 (2.2, 3.7)]. Although the incidence of ARS appeared to increase after some eruptions, especially in areas close (< 26 km) to the volcanoes, we did not find a consistent temporal association between the yearly incidence of ARS and volcanic eruptions when considering the entire observation period. When we analysed shorter time-windows (6 months in the year preceding an eruption), we observed increased ARS incidences in eruptive months, except in 2010. IRRs were increased for centres situated close to volcanoes (< 26 km) in 2001 and 2002.

**Conclusion:**

ARS incident cases increased over the years in populations living around the Nyamulagira and Nyiragongo volcanoes, but we found no consistent evidence for an association between the yearly incidence of ARS and volcanic eruptions or the intensity of SO_2_ emissions, possibly because of interference with man-made events, including massive population displacements caused by insecurity in the area. Nevertheless, some evidence was found for increased incidence of ARS following eruptions, especially in areas close to volcanoes. Assessing personal, ground level exposure to SO_2_ and particulates with adequate controlling for confounding, such as viral and other infections, could clarify the contribution, if any, of volcanic emissions of SO_2_ to the high burden of respiratory diseases in this region.

## Introduction

In areas prone to volcanic eruptions, volcanic degassing leads to the emission of various air pollutants [1], with sulphur dioxide (SO_2_) being one of the most commonly released gases (besides water and carbon dioxide) [2–6]. Volcanic degassing has impacts on the environment (acid rain formation, plant damage) and on climate. In humans, SO_2_ causes irritation of the skin and mucous membranes because of the formation of sulphuric acid (H_2_SO_4_), thus leading to acute or chronic respiratory disorders [7].

In a time series study, Kan et al. [8] showed the daily mortality in Shanghai was related to short-term exposure to outdoor SO_2_, even after adjusting for particulate matter (PM10). Studying health effects of volcanoes, Longo et al. showed increases in respiratory morbidity and mortality due to the Kilauea volcano activity in Hawaii, a sulphurous volcano [9, 10].

**Fig. 1:**
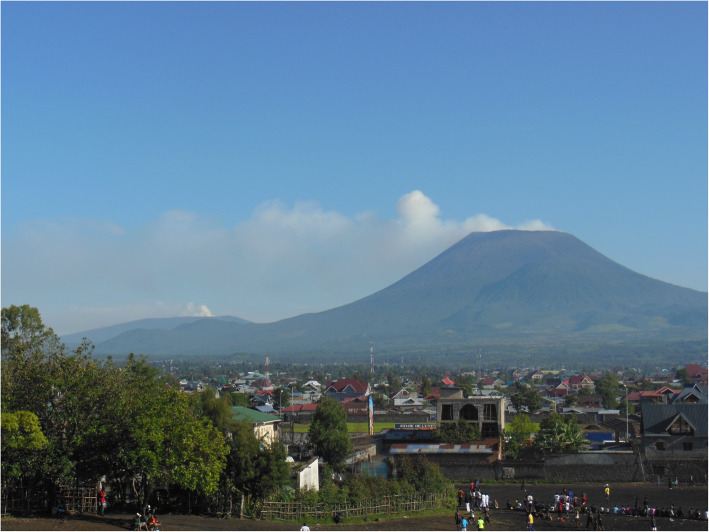
Virunga active volcanoes: Nyamuragira (on the left, in the background) and Nyiragongo (on the right) in the vicinity of the city of Goma (foreground) (©Nicolas d’Oreye)

Nyiragongo and Nyamulagira, located in the Virunga National Park (Fig. 1), are among the most active African volcanoes [11] and more than one million people living in the eastern Democratic Republic of Congo (DRC) are potentially exposed to their hazardous effects. The volcanic plumes from both volcanoes are dispersed over several hundred kilometres in a fairly constant direction, mainly to the west-south-west (266 ± 12 deg) [12]. Nyiragongo (with two historical eruptions, 1977 [13] and 2002 [14]) has the largest persistent lava lake worldwide that produces a SO_2_-rich plume. Its neighbour Nyamulagira (more than 40 eruptions, last in 2012) has an impressive activity average of one eruption every 2–4 years [11]. Most importantly, unlike explosive volcanic eruptions that send particulates and gases into the stratosphere, Nyamulagira’s effusive eruption emits gases and aerosols into the lower troposphere, hence raising concerns for human health [15, 16]. Critically, these volcanoes are located close to a densely populated area, especially the city of Goma with almost one million inhabitants living only 15 to 30 km from the volcanoes [17].

Considering that acute respiratory symptoms (ARS) are most consistently associated with exposure to volcanic degassing [1, 18], we have tested the following hypotheses using routinely collected health data: 1/ volcanic degassing, identified through eruptive emissions of SO_2_ during volcanic eruptions, was associated with an increased incidence of ARS between 2000 and 2010 and 2/ the incidence of ARS decreases with increasing distance to volcanoes.

## Methods

### Data, settings, and study design

In the framework of the GORISK project (Combined use of Ground-Based and Remote Sensing techniques as a tool for volcanic risk and health impact assessment for the Goma region; http://www.ecgs.lu/gorisk/), Michigan Technological University (MTU, USA) provided satellite data retrievals of volcanogenic SO_2_ gas columns determined with the Ozone Monitoring Instrument (OMI). SO_2_ concentrations were expressed in Dobson Units (DU) and then converted into kilotons (one DU is 0.01 mm thick and contains 0.0285 g of SO_2_ per square meter). These remote sensing data give insights about the spatial distribution of the average Nyiragongo and Nyamulagira plumes, calculated between October 2004 to December 2008, which disperse over several hundred kilometers in a fairly constant direction, mainly to the west-southwest (266 ± 12 deg) [12]. More precisely, the data cover the semi-permanent Nyiragongo degassing during the above-mentioned time-period, as well as the degassing emitted during the 2006 Nyamulagira eruption (27 Nov to 5 Dec 2006). The values range from 1.1 to 3.7 DU [12]. In addition, Chalmers university and the Goma Volcano Observatory estimated that the average plume height was 3174 m (± 761) over the time period 2007–2010 [12]. These data were used to select the sampling areas for studying the human health impact of volcanic emissions.

Health data were obtained from the North-Kivu province’s health information system (HIS) organized by the provincial Ministry of Health, with the support of CEMUBAC (NGO of the Université Libre de Bruxelles). The system consists of paper registers with demographic and medical information (diagnosis) for each visit to a health centre or hospital, as recorded by local health workers. Of 150 health centres situated under the plume trajectory, 72 had to be excluded due to data incompleteness and uncertain quality, thus leaving 78 centres for statistical analysis. The presence of ARS was based on the diagnosis recorded by the medical staff. We took into account the diseases diagnosed as acute upper respiratory infections (J00-J06), influenza and pneumonia (J09-J18), other acute lower respiratory infections (J20-J22), and chronic obstructive pulmonary disease with acute lower respiratory infection and chronic obstructive pulmonary disease with (acute) exacerbation (J44.0 and J44.1). Children and adults were included in the same dataset.

### Statistical analysis

We first performed descriptive analyses using STATA V13. We then applied negative binomial regression models. Incidence Rate Ratios (IRR) with 95% confidence intervals (CI) were derived from these models for clusters of health centres defined per (a) their distance to Nyiragongo, (b) each year of the decade 2000–2010 and (c) months following eruptions. The equation of all models had the same form.

$$ Ln\ \left( nb. ARS\right)= intercept+{b}_1.{X}_2+{b}_2.{X}_2+\cdots $$

Number of ARS is the dependent variable and the X_j_’s are the independent variables, characteristics of health centers, and the intercept is the log of the baseline incidence rate. All these characteristics were categorical variables and were transformed into indicators to be included in the models.

Considering the “distance to the volcanoes”, we used the quartiles of the distance to the Nyiragongo volcano to categorize the distance and divided the 78 health centres into four buffers defined a priori by quartiles: (i) within 26 km from the volcanoes; (ii) between 26 km and 48 km; (iii) between 49 km and 102 km; (iv) beyond 102 km. These thresholds correspond to the variations in altitude, which are thereby also taken into account: (i) flat area between Goma and Sake to the west; (ii) the rift escarpment and the high plateau; (iii) downhill from the high plateau to the west of the rift steep slope; (iv) eastern border of the Congo river basin (hillside area).

We then analyzed the number of ARS according to years and according to months following an eruption, for the whole area and within each distance category.

For the long-term temporal analysis (“incidence of ARS by year”), IRR’s were estimated for each year of the decade 2000–2010, with 1999 being the reference year, as no volcanic eruption occurred during that year.

For the short-term temporal analysis (“incidence of ARS by months after eruptive event”), to account for seasonal effects, a period of four to 6 months without any eruption, included within a year before the beginning of the analyzed eruption, was chosen as reference [except for the 2001 Nyamulagira eruption (6 February – 5 May), for which we took 1999 as the reference period because an eruption also occurred in January 2000].

The size of the population at risk (population figures included in the HIS) was introduced as exposure in the models, and to take the clustering into account, we used a model with robust (cluster) standard errors that allow for intragroup correlation.

## Results

Based on data of SO_2_ emissions by eruptions of Nyamulagira and Nyiragongo from 1999 to 2010 published by Bluth and Carn [15] and the Global Volcanism Program website of the Smithsonian Institute (http://volcano.si.edu), the total amounts of SO_2_ (kT) emitted during eruptions varied between 0.093 kT and 4.5 kT (Table 1).

**Table 1:** Total flux of SO2 (kT) emitted during eruptions of the Nyamulagira and Nyiragongo volcanoes between 2000 and 2010

Volcano	Year	Total SO_**2**_ flux mass (kT)
Nyamulagira	2000	0.31
	2001	1.73
Nyiragongo	2002	0.093
Nyamulagira	2002	2.32
	2004	2.6
	2006	4.2
	2010	4.5

Based on health data from the North Kivu province’s Health Information System (HIS), the number of health centres – from dispensaries serving small communities to large hospitals – grew from 456 registered structures in 1999 to 812 in 2010. At the provincial scale, malaria was the more frequently recorded disease (32.3% of total cases, about 8.4 million cases) for the 10-year time period, followed by ARS (12.2% of total cases, about 3.2 million cases).

**Fig. 2:**
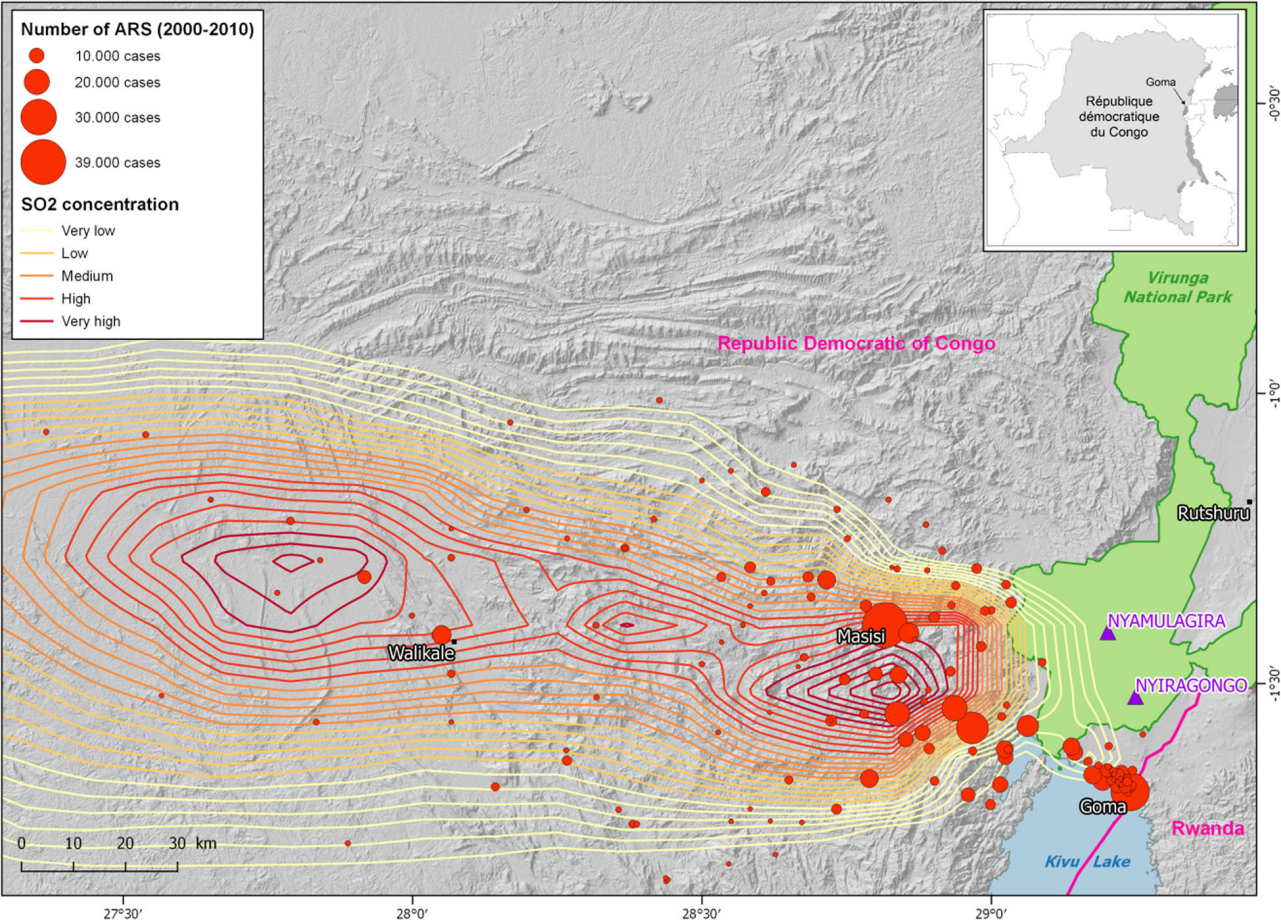
Distribution of the total number of cases of acute respiratory symptoms (ARS), from 1999 to 2010, over the studied area. The isopleths of volcanogenic SO_2_ concentrations are based on satellite data obtained using the Ozone Monitoring Instrument (OMI) and dominant wind directions [16]

As shown in Fig. 2, showing the location of the health centres included in the HIS from 1999 onwards, more ARS cases were reported close to Goma city and in the “hauts plateaux” (highlands at 1500 to 2500 m above sea level (asl)).

**Fig. 3:**
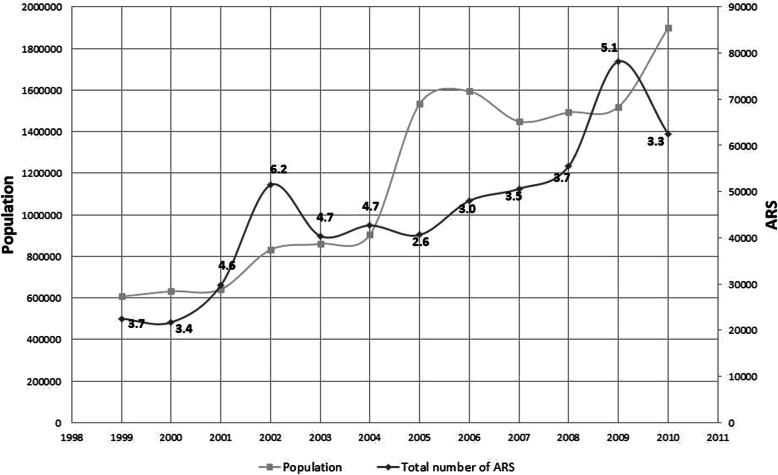
Population and acute respiratory symptoms (ARS) registered in the targeted health centres between 1999 and 2010: Population covered by 78 health centres and total number of ARS cases registered in these health centres in each year, from 1999 to 2010. Numbers in the graph indicate the yearly incidence of ARS in %

We estimated the yearly population incidence of ARS (i.e. the number of new cases of acute respiratory symptoms registered per year over the number of residents during that year) for the whole study area (Fig. 3). Over the period of interest, the total population in the region more than doubled. The average yearly incidence of ARS was 4.0%, but with substantial fluctuations from year to year, between an apparent low incidence (2.6%) in 2005 and apparent peaks in 2002 (6.2%) and 2009 (5.1%).

**Fig. 4:**
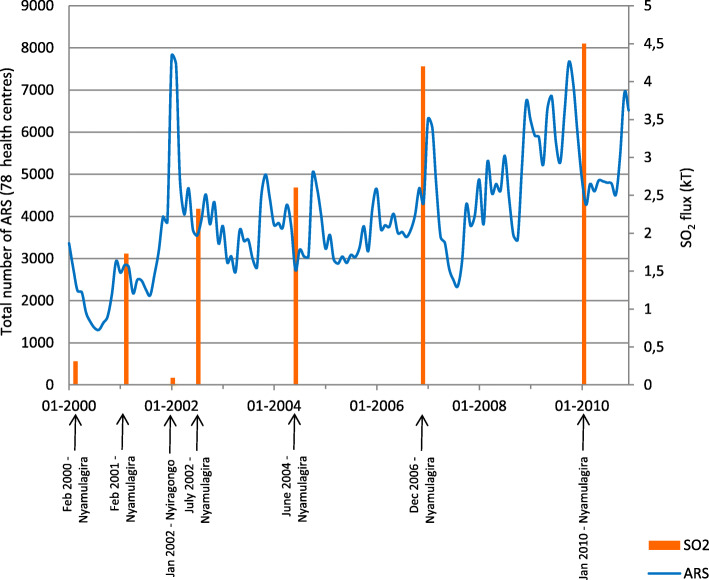
Number of new cases of acute respiratory symptoms (ARS) registered per month in the 78 selected health centres between 2000 and 2010 (blue line). Each eruption (orange columns) is sized according to the total SO_2_ discharged throughout the eruptive event

Figure 4 depicts the years of volcanic eruption occurrence (represented with their total SO_2_ discharge) and the numbers of ARS cases registered per month. The general trend indicates a constant increase of reported ARS cases, from a monthly average number of about 2000 cases per month in 2000 to 6255 cases per month in 2009 and 5100 in 2010. Seasonal peaks are observed at the end/beginning of each year, corresponding to the short rainy season. The total numbers of reported ARS cases were particularly high in 2002 (*n* = 56,190) and 2009 (*n* = 75,061), compared to the period between 2003 and 2007 with yearly numbers of cases fluctuating around 44,000.

The peaks in ARS did not coincide with the eruptions, and higher total emissions of SO_2_ were not associated with higher incidences of ARS. A huge peak of ARS did follow the Nyiragongo eruption of 17th January 2002 with 7800 and 7600 cases reported in January and February, respectively, vs. 4870 cases in March 2002. However, the total number of registered diseases also peaked in that period, including for conditions that were unlikely to be linked to volcanic degassing (e.g. 32,382 cases of malaria in February 2002 vs 16,413 cases of malaria in March 2002).

**Fig. 5:**
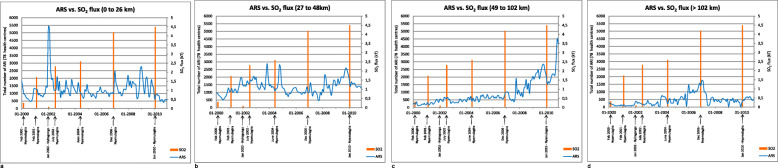
(**a**, **b**, **c** and **d**)- ARS and SO_2_ flux between 2000 and 2010, according to the distance to the volcanoes. Number of ARS cases registered per month in the selected health centres between 2000 and 2010 (blue line), separated according to distance to the volcanoes. Each eruption (orange columns) is sized per the total SO_2_ discharged registered throughout the eruptive event

In the area close (< 26 km) to the volcanoes (Fig. 5a), the number of ARS cases remained relatively stable over the period, apart from the peak after the 2002 Nyiragongo eruption. At distances between 27 and 48 km (Fig. 5b) and between 49 km and 102 km (Fig. 5c), no relationships were apparent between volcanic events and number of ARS cases. At further distances (more than 102 km), the trend appeared stable, apart from some increase in the 2005–2007 period (Fig. 5d).

We then analysed the temporal variations in the incidence of ARS by calculating incidence rate ratios (IRR) with their 95% confidence intervals (95% CI).

We considered long-term (over a decade) yearly trends in the incidence of ARS, taking 1999 as a reference (Table 2), and then short-term (monthly) changes in relation to volcanic eruptions, taking relevant monthly periods prior to specific eruptions as references (Table 3). We did this for the whole region and also for each of the four areas defined by proximity to the volcanoes.

**Table 2:** Incidence Rate Ratios (IRR) and 95% confidence intervals of acute respiratory symptoms (ARS) for each year (reference = 1999) for all health facilities and according to their distance to Nyiragongo. Values of IRR in bold reflect significant increases compared to 1999

	≤ 26 km	27 to 48 km	49 to 102 km	> 102 km	Total
Year	IRR (IC 95%)	IRR (IC 95%)	IRR (IC 95%)	IRR (IC 95%)	IRR (IC 95%)
1999	1	1	1	1	1
2000	0.8 [0.6–1.2]	1.2 [0.9–1.5]	0.8 [0.6–1.0]	0.8 [0.7–1.1]	0.9 [0.8–1.1]
2001	1.0 [0.6–1.6]	**1.8 [1.4–2.4]**	0.9 [0.6–1.4]	0.8 [0.6–1.0]	1.1 [0.9–1.4]
2002	**2.6 [1.2–5.6]**	**2.3 [1.8–3.0]**	1.0 [0.7–1.5]	1.5 [0.9–2.5]	**1.8 [1.4–2.4]**
2003	1.5 [0.7–3.1]	**2.1 [1.5–2.9]**	1.0 [0.8–1.4]	**1.6 [1.2–2.2]**	**1.5 [1.2–1.9]**
2004	1.4 [0.7–2.8]	**2.2 [1.6–3.0]**	**1.3 [1.0–1.7]**	**2.1 [1.0–4.4]**	**1.7 [1.3–2.3]**
2005	1.6 [0.8–3.2]	**1.5 [1.0–2.2]**	**1.5 [1.0–2.4]**	**2.4 [1.5–3.6]**	**1.7 [1.4–2.2]**
2006	1.6 [0.8–3.1]	**1.5 [1.1–2.0]**	**1.6 [1.1–2.3]**	**4.2 [2.5–7.1]**	**2.2 [1.6–3.0]**
2007	**2.2 [1.1–4.4]**	**1.6 [1.2–2.0]**	**2.0 [1.2–3.2]**	**3.7 [2.5–5.4]**	**2.3 [1.8–3.0]**
2008	**4.0 [1.5–11.0]**	**2.5 [1.7–3.6]**	**2.1 [1.3–3.5]**	**2.2 [1.7–2.9]**	**2.7 [1.8–3.9]**
2009	**2.5 [1.0–6.3]**	**2.8 [1.9–4.2]**	**3.9 [2.5–5.9]**	**1.9 [1.4–2.5]**	**2.8 [2.2–3.7]**
2010	0.9 [0.4–2.0]	**2.4 [1.5–3.7]**	**3.8 [2.4–6.0]**	**2.3 [1.7–3.0]**	**2.4 [1.8–3.3]**

**Table 3:** Incidence Rate Ratios (IRR) and 95% confidence intervals of acute respiratory symptoms (ARS) for each eruptive month(s) (reference = previous year’s same month(s)) for all health facilities and according to their distance from Nyiragongo. Values of n concern the numbers of health centres and values in italics indicate the IRR with significant *p* values for the comparison with the overall IRR

Eruption	Date	IRR ≤ 26 km (*n* = 17)	IRR > 102 km (*n* = 21)	IRR overall (*n* = 78)
Nyamulagira 2001, 6th Feb to 5th May	Jan - May 1999	1	1	1
	Dec 2000	1.8 [0.9–3.7]	*1.0 [0.6–1.7]*	1.4 [1.1–1.9]
	Jan 2001	1.9 [1.0–3.5]	*0.9 [0.7–1.3]*	1.3 [1.0–1.6]
	Feb - Apr 2001	1.4 [0.8–2.6]	*0.9 [0.6–1.2]*	1.2 [1.0–1.5]
	May 2001	1.4 [0.7–2.8]	0.8 [0.5–1.1]	1.2 [1.0–1.5]
Nyiragongo 2002, 17th Jan	Nov 2000 - Feb 2001	1	1	1
	Nov 2001	1.3 [0.9–2.0]	*0.9 [0.5–1.4]*	1.3 [1.1–1.5]
	Dec 2001	1.2 [0.8–1.8]	*1.0 [0.7–1.5]*	1.3 [1.1–1.5]
	Jan 2002	4.9 [2.3-10.7]	1.9 [1.2–3.3]	2.1 [1.4–3.2]
	Feb 2002	5.8 [2.4–13.9]	2.1 [1.2–3.7]	2.3 [1.5–3.6]
Nyamulagira 2002, July to Sept	May - Oct 2001	1	1	1
	May 2002	2.8 [1.4–5.4]	2.1 [1.2–3.8]	1.7 [1.3–2.2]
	June 2002	1.6 [0.9–3.1]	1.8 [1.0–3.2]	1.4 [1.1–1.7]
	July - Sept 2002	2.6 [0.9–7.2]	1.5 [0.8–2.9]	1.6 [1.2–2.3]
	Oct 2002	1.8 [1.0–3.2]	1.8 [1. 0–3.1]	1.5 [1.2–1.8]
Nyamulagira 2006, 27th Nov to 5th Dec	Oct 2005 - Feb 2006	1	1	1
	Oct 2006	*0.9 [0.7–1.1]*	1.4 [1.0–1.9]	1.1 [0.9–1.3]
	Nov 2006	*1.1 [0.9–1.4]*	1.6 [1.1–2.4]	1.3 [1.0–1.6]
	Dec 2006	1.1 [0.9–1.5]	1.4 [1.0–2.0]	1.3 [1.0–1.7]
	Jan 2007	2.1 [1.6–2.6]	2.2 [1.4–3.3]	1.8 [1.4–2.4]
	Feb 2007	1.5 [1.0–2.2]	1.9 [1.3–2.8]	1.7 [1.3–2.1]
Nyamulagira 2010, 2nd to 27th Jan	Nov 2008 - Feb 2009	1	1	1
	Nov 2009	0.4 [0.2–0.7]	0.8 [0.6–1.1]	1.1 [0.8–1.5]
	Dec 2009	0.3 [0.2–0.7]	0.8 [0.6–1.0]	0.9 [0.7–1.3]
	Jan 2010	0.2 [0.1–0.3]	0.8 [0.6–1.0]	0.7 [0.6–1.1]
	Feb 2010	0.3 [0.2–0.4]	0.9 [0.7–1.3]	0.7 [0.5–0.9]

Over the decade, a consistent, almost three-fold, increase in the overall incidence of ARS was observed between 2000 [IRR 0.9 (95% CI 0.8–1.1)] and 2009 ([IRR 2.8 (95%CI 2.2–3.7)] (Table 2). This occurred in the various areas. However, in 2010, the IRR of the area < 26 km fell down to 0.9 (95%CI 0.4–2.0). No consistent increases in monthly incidences of ARS were found during or following volcanic eruptions, neither when compared with the reference year (1999; Table 2), nor when compared with the months preceding the eruptions or with the same months in the preceding year (Table 3).

Nevertheless, the Nyiragongo eruption of January 2002, with low SO_2_ emission (0.093 kT), seemed to have a great impact, i.e. a pronounced increase in the incidence of ARS, compared to January 2001, especially in the area close (i.e. < 26 km) to the volcano (IRR: 4.9). The 2006 Nyamulagira eruption lasted a short period, with a great SO_2_ flux degassing. On the contrary, the Nyamulagira eruption of January 2010, with high SO_2_ emission (4.5 kT), was accompanied and followed by a marked decrease in ARS incidence compared to January 2009, in the vicinity of the volcano (IRR: 0.2) (Table 3).

## Discussion

Although our study found ARS to be the second most common cause of medical visits (after malaria) in areas around the volcanoes such as the city of Goma, as well as in the highlands between 1500 to 2500 m (asl), we found little if any convincing associations between the incidence of ARS (at least as assessed in our study) and the occurrence or intensity of the Nyamulagira and/or Nyiragongo volcanic eruptions (as assessed by SO_2_ emissions) during the observation period (2000–2010). However, the absence of a clear associations does not allow us to conclude that the volcanic eruptions did not affect respiratory health in the area.

Our study is the first to have investigated the possible health effects of volcanic eruptions with pronounced degassing of SO_2_ in the region. A major strength of our study is the use of morbidity data collected over a whole decade among several hundred thousand inhabitants of a large area. Nevertheless, our findings suggest that the impact of volcanic degassing of the Virunga volcanoes on human respiratory health is not straightforward. Similarly to our findings, Tam et al. failed to demonstrate (in a small community of 1957 people) an association between respiratory health problems, such as asthma, chronic persistent wheeze or bronchitis, and chronic exposure to acid fog from a high degassing volcano, the Kilauea on the Island of Hawaii [19]. In a study conducted in the Azores, the authors highlighted that the volcanogenic air pollutants (i.e. non-eruptive active volcanism) have a high potential to cause lung injury in the long term [20], but detailed studies of chronic respiratory morbidity are difficult to perform in an under-resourced region such as eastern DRC. In Japan, Iwasawa [21] found that short-term acute exposure concentrations contribute more to irritation symptoms than average exposure concentrations. Indeed, the exposure duration might not be a determining factor because responses occur very rapidly, within the first minutes following the beginning of inhalation; apparently, further exposure does not increase effects [22]. However, a review by Gudmundsson suggested that acute and chronic respiratory effects varied with the ash composition, which differed from volcano to volcano, as well as from eruption to eruption [23]. Thus, Ishigami et al. reported a significant exposure-response relationship between volcanic SO_2_ and ARS over time among 611 health volunteers newly arriving after 2 years of the Miyakejima volcano eruption in Japan [24]. Wakisaka et al. found an association between monthly reported clinical respiratory cases and levels of SO_2_ rather than total suspended particles, after adjusting for seasonality [25]. This suggests not only a possible synergistic effect between SO_2_ and suspended particles [26], but also the fact that rainy season could potentiate SO_2_ toxicity even though no adjustment for meteorological factors was performed.

The Nyiragongo eruption of 2002, its worst eruption in living memory, and the associated high peak in the incidence of ARS (Figs. 4 and 5a) deserves a detailed discussion. This catastrophic event, with lava reaching the city of Goma, had serious short-term and long-term impacts on the city’s economy and its environment (vegetation and water) [27]. Its public health consequences have been addressed in a report to the WHO, which described a high number of consultations for eye and respiratory problems in the days following the eruption. The peak of attendances could hardly be linked to the volcanic degassing emitted during this eruption (17th January) because the estimated total SO_2_ emission (15–48 kT; Table 1) [28] was a magnitude lower than the typical daily discharge observed during an eruption of the neighbouring Nyamulagira. The 23rd to 26th January morbidity peak was more likely linked to the collapse of the crater floor which occurred during the 22nd January’s night. Then, hot ash fell over the village of Rusayo (8 km SW of the summit) and accumulated to a 10 cm-thick ash layer, whereas lighter ash fell over Goma and Gisenyi [29]. However, as Horwell underlines, the incidence of acute respiratory symptoms (e.g. asthma, bronchitis) varies greatly after ashfalls, from very few, if any, reported cases to population outbreaks of asthma [30]. It is, therefore, difficult to conclude to any relationship. Other gases may have been partly responsible for this peak: abnormal smells of hydrocarbon gas and numerous gas bursts were reported to have occurred in Goma after the eruption and the lava flow invasion, principally on January 20th to 22nd [29, 31]. However, although reports indicate that air quality was affected by smoke and particulate matter in the first days after the eruption, it soon returned to normal [31].

Moreover, in the HIS database, a peak of attendances was not only observed for respiratory diseases. In fact, fearing a cholera epidemic, a programme of free health care and drug supply was implemented to support the primary health centres in Goma for a six-week period following the 17th January eruption, followed by another six-week period with reduced prices (0.2 US$ instead of 1 US$ for ambulatory care services, including drugs) [32]. As a consequence, the epidemiological surveillance programme showed a large increase in total attendances at the two hospitals and 18 functioning primary health care centres after the eruption. This apparent increase in overall morbidity must be attributed to the free treatment offered in the wake of the 2002 Nyiragongo eruption. Attendances dramatically decreased, when the free health care programme stopped (1st of March) [31], notably because not only people living within the Goma health district had come to be freely treated, but also those living in bordering health districts [32]. Thus, a higher proportion of patients came from neighbouring districts in January (14.6%) and February (18.6%) than in March (13.6%).

Looking at the Nyamulagira eruptions, the risk of developing ARS during the period of the 2006 event appeared to be high, and increasing with the distance to the volcanoes. Tremendous amounts of volcanic discharges during this short eruption might have quickly travelled westwards [33–36]. In addition, this area is at a high altitude and this factor could also contribute to the higher IRR registered following the November 2006 eruption. As Delmelle explains [37], the local topography exerts a strong influence on plume dispersal, and hilltops are particularly prone to fumigation and thus, to high ambient SO_2_ levels. On the contrary, it could also explain the low IRR obtained for the population living at lower altitudes less than 25 km from the volcanoes. However, more detailed analyses would be required. Our findings could strengthen the role played by anthropogenic factors, as well as by climate, in the amplification of exposure due to volcanic degassing. Indeed, outside of the city of Goma, the degree of poverty is high, which means that poverty and its covariates (such as malnutrition, use of biomass fuel etc.) could be predictors of respiratory diseases and could constitute potential confounding. This has been partially controlled by comparison with the reference year (1999) but could be investigated in more details.

In contrast, the high IRRs registered between 2008 and 2009 are unlikely to have resulted from volcanic emissions, as these two years were free of eruptions. These high IRRs may be attributed to the insecurity that prevailed from mid-2008 to end-2009, leading groups of internally displaced persons (IDPs) to settle around Goma. As observed through the reported ARS cases, during this period, an increased number of people attended health centres outside their area of origin. In addition, during these periods of insecurity, international NGOs started to provide free medical care; anecdotal reports mention an extra work load because many people used health care services, even though their physical or mental conditions did not always require any treatment [32]. The high IRRs reported in 2008 and 2009 could, therefore, be due to the massive presence of IDPs around Goma fleeing from conflict situations. As this security crisis phase was used as the reference period (November 2008 to February 2009), it could in turn explain the “low” ARS IRR of January 2010. This illustrates the complexity of investigating the health impact of eruptive events since our IRRs were sometimes affected by inappropriate reference periods.

In our research, because of lack of data, the possible effects of particulates were not considered, although several studies [38] identify particulate matter as a major cause of ARS, especially in developing countries, where the main cooking fuel remains wood and charcoal. In addition, our study did not take into consideration the weather variations throughout the year, nor the day and night temperatures fluctuations, which can be strong compared to the relatively stable annual temperatures. Whilst temperatures do not have a direct impact on ARS, people do change their behaviour according to temperature and weather.

We recognize that uncertain quality and completeness of the health data – from collection to encoding – might represent a significant limitation of our study. Although the increased number of registered ARS cases possibly reflects an improvement of the HIS and data collection over time, routine registration was not accurate in every health centre. Moreover, the denominator (population covered by the health centre) was imprecise due to difficult local conditions, such as internal migration because of war and violence [39]. Consequently, we strongly recommend improving the data collection system and monitoring its quality to allow research based on reliable information. Besides, not all people have the capacity to go to health centres when health problems occur, mainly because they lack resources or because of long distances. This leads to incomplete morbidity data in the HIS used in this study. A household-based approach would provide complementary data. This approach should also be associated with actual measurements of the various components of the ground level volcanic degassing in the field. The long-term perspective would involve the combination of SO_2_ and PM measurements with regularly collected meteorological data, not available today. Lastly, local institutions assessing risks associated with the volcanoes and providing precautionary options remain weak. The study of Cuoco et al. [40] following the 2010 Nyamulagira eruption suggested that the impact on the environment – through contaminated drinking water (exposure to toxic metals and chemical species) – was substantial and could have consequences in terms of public health, such as renal, gastrointestinal, cardiovascular and neurological diseases, as well as birth defects, thyroid disease, cancers, and osteoporosis. However, as exposure to pollutants is largely beyond the control of individuals, it requires action from the public authorities [41].

## Conclusions

Our large-scale study was not able to document that the volcanic eruptions of the Nyamulagira and Nyiragongo volcanoes in the east of the DRC had a serious or measurable impact on respiratory health among surrounding populations, but the subject deserves further prospective study given the known effects of volcanic degassing on respiratory health. With improved exposure assessment, systematic registration of symptoms and diseases, and the availability of baseline or control data, the outputs of such a study could reveal an impact on respiratory health in this region. Our study was based on SO_2_ measurements made using the OMI sensor that was available during the investigated period. In the perspective of a new study, it would be interesting to make use of the TROPOMI sensor of the European Copernicus programme, whose performances are very promising for this type of study. Future studies in the area of risk assessment should contribute to provide appropriate recommendations (water treatment, early warning system, etc.) and lead to an improved assessment of the impact of volcanic activity on human health.

## Data Availability

The data will be made available by the principal investigator upon reasonable request and according the agreement established with CEMUBAC.
